# Detection of Soil Nitrogen Using Near Infrared Sensors Based on Soil Pretreatment and Algorithms

**DOI:** 10.3390/s17051102

**Published:** 2017-05-11

**Authors:** Pengcheng Nie, Tao Dong, Yong He, Fangfang Qu

**Affiliations:** 1 College of Biosystems Engineering and Food Science, Zhejiang University, Hangzhou 310058, China; npc2012@zju.edu.cn (P.N.); 21613052@zju.edu.cn (T.D.); quff1128@163.com (F.Q.); 2 State Key Laboratory of Modern Optical Instrumentation, Zhejiang University, Hangzhou 310058, China

**Keywords:** nitrogen, near-infrared sensor, soil pretreatment, PLS, UVE, CARS, LS-SVM

## Abstract

Soil nitrogen content is one of the important growth nutrient parameters of crops. It is a prerequisite for scientific fertilization to accurately grasp soil nutrient information in precision agriculture. The information about nutrients such as nitrogen in the soil can be obtained quickly by using a near-infrared sensor. The data can be analyzed in the detection process, which is nondestructive and non-polluting. In order to investigate the effect of soil pretreatment on nitrogen content by near infrared sensor, 16 nitrogen concentrations were mixed with soil and the soil samples were divided into three groups with different pretreatment. The first group of soil samples with strict pretreatment were dried, ground, sieved and pressed. The second group of soil samples were dried and ground. The third group of soil samples were simply dried. Three linear different modeling methods are used to analyze the spectrum, including partial least squares (PLS), uninformative variable elimination (UVE), competitive adaptive reweighted algorithm (CARS). The model of nonlinear partial least squares which supports vector machine (LS-SVM) is also used to analyze the soil reflectance spectrum. The results show that the soil samples with strict pretreatment have the best accuracy in predicting nitrogen content by near-infrared sensor, and the pretreatment method is suitable for practical application.

## 1. Introduction

The soil, whose main role is to provide nutrients in the process of plant growth [1,2], is the foundation and an important part of agriculture. Nitrogen and other nutrients in farmland are important factors affecting crop growth. It is very important to obtain the soil nitrogen content information quickly and reasonably for proper fertilization and agricultural production [3]. However, the traditional chemical testing method called “soil testing and fertilization” has the disadvantages of taking a long time, and being a complex and high cost process, which greatly limits the detection of soil nutrients [4]. 

Near-infrared sensors applied to the detection of soil nitrogen can quickly obtain information such as nitrogen nutrient levels in the soil and realize data analysis and the detection process is non-destructive and pollution-free [5,6]. In recent years, many scholars have used near infrared sensors to study the nitrogen content in the soil. Mouazen et al. compared the PLS and BPNN modeling methods on a visible near-infrared sensor, and found that the BPNN method was superior to the PLS modeling method in detecting soil organic nitrogen [7]. Kuang et al. collected soil samples from five farms and detected soil organic carbon and inorganic nitrogen by a near infrared sensor, finding that organic carbon RPD (residual prediction deviation) were (2.66–3.39) and inorganic nitrogen RPD were (2.85–3.45) [8]. Shi used a multiple linear regression method to estimate organic nitrogen content in soils based on visible/near infrared (NIR) spectra, and proposed the 1450, 1850, 2250, 2330 and 2430 nm bands as the characteristic bands for this purpose [9]. Cecillon et al. used near-infrared sensor (NIR) techniques and estimated organic matter in the soil and extracted the relevant characteristic wavelengths based on principal component analysis and genetic algorithms [10]. He et al. used a near-infrared sensor to detect N, P, K, organic matter and pH in the soil. It was proved that there was a significant correlation between the reflectance spectra of soil and nitrogen, and the near infrared sensor could predict the soil nitrogen and organic matter and other nutrients [11,12,13]. Li took three parts of soil, including soil surface (0~30 cm), soil interlayer (30~48 cm) and soil bottom layer (48~60 cm) and analyzed the absorption spectrum characteristics of soil samples at different levels and the variation of soil water and nitrogen levels. The results showed that the method had obvious advantages in the prediction of soil nitrogen content [14].

Near-infrared sensors have been widely used in soil nitrogen detection, and relevant scholars have studied the characteristic bands, modeling methods and other aspects. However, in practice, the nitrogen content detection accuracy is not high when the soil is not pretreated well because of the soil water content [15], soil particle size and soil surface roughness [16]. Hernandez, et al. investigated the effect of soil particle size and soil moisture content on soil spectral properties. It was found that if the soil particle size is too large or too small it would affect the accuracy of near-infrared sensors to predict soil organic nitrogen, and the NIR prediction results were not ideal when the soil water content was heavy [17]. Wang studied the soil moisture by detecting the effect of soil organic matter with different water contents using a near infrared sensor. The results showed that the water would mask the characteristic wavelength bands of the soil organic matter and could interfere with the detection of soil organic matter [18]. Zeng et al. studied the effect of water on the content of organic matter detection in purple soil by near infrared spectroscopy. The genetic algorithm, two polynomial fittings and PLS were used to process the spectral data. The results showed that the proposed method could effectively eliminate the influence of purple soil moisture on the prediction of the organic matter content [19]. Zhu used PLS to study the effects of soil moisture and soil particle size on total soil nitrogen detection. The experimental results showed that the smaller the soil water content was and the smaller the soil particles was and the better the accuracy and effectiveness of the detection were [20,21]. However, there are relatively few studies on the strict pretreatment of soil. Soil pretreatment can not only improve the accuracy in detecting soil nitrogen content by near infrared reflectance sensor, but also has a good scientific and practical value.

## 2. Materials and Methods

### 2.1. Sample Preparation

**Figure 1:**
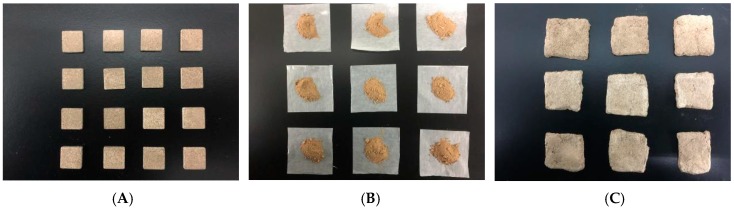
(**A**) Strict pretreatment soil samples; (**B**) grinding and drying soil samples; (**C**) drying soil samples.

Soil samples in this experiment are pure natural soils from Hui Zhuang Agricultural Development Company in Huainan City, Anhui Province, China. The soil texture after artificial screening was fine. Nitrogen solutions with concentrations of 0%, 2%, 4%, 6%, 8%, 10%, 14%, 16%, 18%, 20%, 22%, 24%, 26%, 28%, 30% were prepared for the experiments. The soil samples were divided into three equal groups and each group had 16 parts. First, solutions of different concentration and soil are fully mixed and stirred. Second, the three groups of soil samples are dried at 80 °C for 8 h. Third, the first group of soil samples are pretreated by grinding with 80 mesh (160 μm) sieve screening and pressing them into 10 mm × 10 mm samples of 2 mm thickness under a the pressure of 10 MPa using a bench press machine (Figure 1A). The second group of soil samples were dried and ground as shown in Figure 1B. The third group of soil samples was dried without any pretreatment, as shown in Figure 1C. There are 10 samples of each soil sample of each concentration. 

### 2.2. Spectrometric Determination

The spectral reflectance information of the three groups of soil samples was collected by a spectrometer. This experiment used a near infrared optical spectrum instrument from Wuling Company (Shanghai, China). The spectral range, standard rate and scanning times can be set based on the experimental requirements. The spectral acquisition range was from 900 nm to 1700 nm. The near infrared optical spectrum instrument can collect the light intensity, reflection and absorption information from soil. Each spectral acquisition is set up with 400 points and a spectrum was obtained by averaging three scans. 

### 2.3. Data Analysis

The principle of near infrared sensor is the absorption of the molecule frequency doubling. NIR sensors can detect the energy not absorbed by the material due to the chemical composition. However, at the same time, it can also be affected by the surface texture, density and uneven distribution of internal components, which makes it very difficult for all the redundant information of the spectral information to be eliminated, such as the overlap of the spectral information, the large amount of noise and the sample background [22]. If these data were involved in modeling, it not only would result in a large amount of computation and model complexity, but also would influence the results’ accuracy. Therefore, in order to achieve the purpose of qualitative or quantitative analysis of complex mixtures, it is necessary to extract and analyze the weak chemical information in the spectral analysis by chemometrics [23]. In this paper, three different liner data processing methods and one nonlinear data method were used to model and analyze the reflectance spectra. 

#### 2.3.1. Partial Least Squares Method

Partial least squares regression is the most commonly used chemometrics modeling method in spectral data analysis [24,25]. The selection of the main factor is directly related to the actual prediction ability of the model when using this analysis method. If the number of main factors is too small, the spectral information of the sample cannot be fully expressed. On the contrary, if the number of main factors is too large, the increase of noise would reduce the prediction ability. Therefore, it is very important to establish the prediction model by taking into account the spectral matrix *X* and the physical and chemical value of *Y* in order to obtain the latent variables and reduce the influence of the useless variables. The advantage of this method is that it can be applied to complex analysis system and small sample multivariate data analysis [26].

#### 2.3.2. No Information Variable Elimination Method

The no information variable elimination method (Elimination of Uninformative Variables, or UVE) was originally proposed by Centner et al. [27]. Teófilo proposed combination algorithms on the basis of UVE [28]. The UVE algorithm establishes a variable selection method based on the PLS regression coefficients. It adds a small random variable matrix to the PLS model, and then establishes the PLS model based on cross validation [29]. It calculates the mean of each variable coefficient and the quotient of the standard deviation as the value of stability. Finally, it compares with the value of the stability of the random variable matrix and the removal is considered to be the same as random variables. The algorithm steps are as follows [30]:

(1) Regress the calibration matrix *X* (*n* × *m*) and the density matrix *Y* (*n* × 1) by PLS to determine the optimal principal component number, where *n* is the number of samples in the calibration set and *m* is the number of wavelengths in the spectrum.
(2) Produce a noise matrix *R* (*n* × *m*) by man-made and combine *X* and *R* into a new matrix *XR* (*n* × 2*m*). The first m columns of the matrix are the spectral matrix and the m columns are the noise matrices.
(3) Regress matrix *XR* (*n* × 2*m*) and Matrix *Y* (*n* × 1) to obtain the coefficient matrix *B* (*n* × 2*m*) composed of *n* sets of regression coefficient vectors by eliminating one sample every time.
(4) Calculate the standard deviations (1 × 2*m*) and the average value *m* (1 × 2*m*) of *B* (*n* × 2*m*) and the reliability of each variable. The mathematical expression of reliability is as follows. *Ci* = *m*(*i*)/*s*(*i*), *i* = 1, 2..., 2*m*.
(5) Take the maximum absolute value of C in the interval [*m* × 2*m*] Cmax = max [abs (C)].
(6) Remove the variable C, (C = Cmax) in the spectral matrix *X* from the interval [1 × *m*].
(7) The remaining variables are grouped into a new matrix XUVE of the optimization variables obtained by filtering the UVE variable.

#### 2.3.3. Competitive Adaptive Weighting Method

The competitive adaptive weighted algorithm method, imitating the evolution of “survival of the fittest” principle, phases out of the invariable wavelengths [31,32]. It uses Monte Carlo sampling or random sampling method to select a part of the sample from the calibration set samples for PLS modeling and repeats this process for hundreds of iterations. The algorithm steps are as follows:

(1) Collect the samples for n times by using Monte Carlo method. Randomly select a certain proportion of samples each time from the sample set as the calibration set.
(2) Establish the PLS regression model by using the extracted spectral matrix *X* (*n* × *m*) and the concentration matrix *Y* (*n* × 1)
(3) Use the exponentially decreasing function (EDF) to remove the wavelength points with small absolute value of regression coefficient.
(4) Collect samples for *i* times and determine the retention rate of wavelength points where *a* and *k* are constants according to EDF calculation formula. It is calculated as follows:
  (1) $$a={\left(\frac{m}{2}\right)}^{\frac{1}{N-1}},k=\frac{\ln\left(\frac{m}{2}\right)}{N-1}$$

It can be seen from the above equation, when sampling the first time, that is, *ri* = 1, m variables are involved in the modeling.

In the process of *N* sampling, we retain the PLS regression model with the absolute value of the PLS regression coefficient and establish the PLS regression model with the selected wavelength variable. Calculate the RMSECV value of the model and select the minimum RMSECV value corresponding to the subset of variables for the optimal subset of variables. 

#### 2.3.4. Partial Least Squares Support Vector Machine Method

Partial least squares support vector machine is a new promising classification technique proposed in 1995 by the Bell Laboratory Research Group of AT&T led by Vapnik [33]. LS-SVM is a kind of learning pattern recognition method based on the theory of statistics, mainly used in the field of pattern recognition. The core idea of LS-SVM is to find an optimal classification hyperplane satisfying the classification requirements. The basic idea of LS-SVM is to map the nonlinear training data to a higher dimensional feature space (Hilbert space) and to find a hyperplane where positive and negative examples both edge isolation between the maximum in the high dimensional feature space [34]. The appearance of LS-SVM effectively solves the problems of traditional neural network, such as the selection of results, the local minimum and over fitting. It shows very impressive performance for small samples, and nonlinear and high dimensional data such as machine learning problems, so it has been widely used in pattern recognition, data mining and other fields [35].

## 3. Results and Discussion

### 3.1. Near Infrared Spectrum Analysis

**Figure 2:**
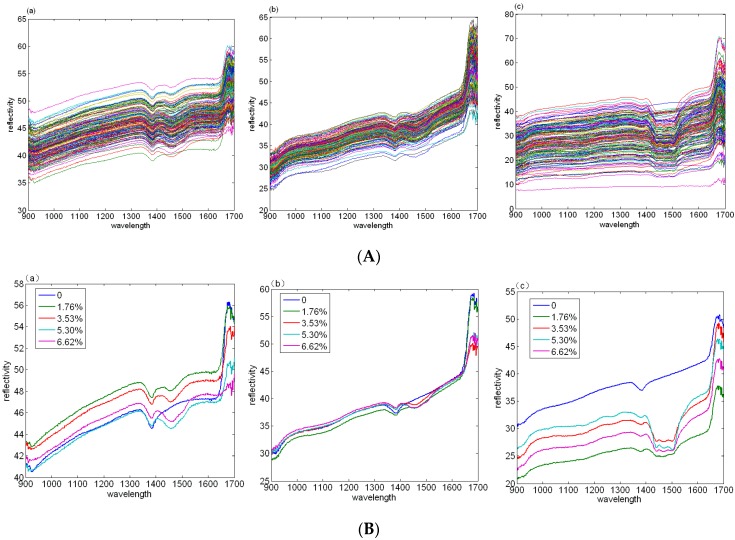
(**A**) three near infrared reflectance spectra of different concentrations of soil nitrogen; (**B**) five different soil nitrogen content samples from the near infrared reflectance spectra of the three groups.

The spectral nitrogen concentration information of the three different pretreatments of the soil samples collected by a Wuling near infrared portable optical spectrum instrument in this experiment is shown in Figure 2A. In this paper, the spectral reflectance image is taken as the research object. The five kinds of soil nitrogen content are selected from the soil samples of the three groups shown in the Figure 2B, respectively. Their concentrations are 0%, 1.76%, 3.53%, 5.30%, and 6.62%, respectively. 

According to the reflectance characteristics of soil samples with different nitrogen concentrations, in the first experiments, the spectral reflectance near the 925 nm band and 1410 nm band decreases significantly when the soil is not added with (CO(NH_2_)_2_), which shows that the absorption of nitrogen in this band is sensitive. With the increase of urea concentration, the spectral reflectance decreases gradually in the 1485 nm band and 1640 nm band, which shows that the nitrogen is sensitive to this band and gradually increases with the increase of nitrogen concentration. The curve trend of the second group of experimental samples is basically same as that of the first group, but the curve noise and the curve smoothness are worse than those of the first group and there was no significant different reflectance between the five groups with different concentrations of nitrogen. The reflectance effects of the third group is significantly worse than that of the former two groups and its absorption is not obvious at 925 nm and 1410 nm. With the increase of nitrogen concentration, the spectral reflectance from 1420 nm to 1475 nm drops in the characteristic band from 1475 nm to 1520 nm and fluctuates significantly. The relationship between spectral reflectance and soil nitrogen is not good in the third group.

### 3.2. Linear Modeling and Analysis

**Figure 3:**
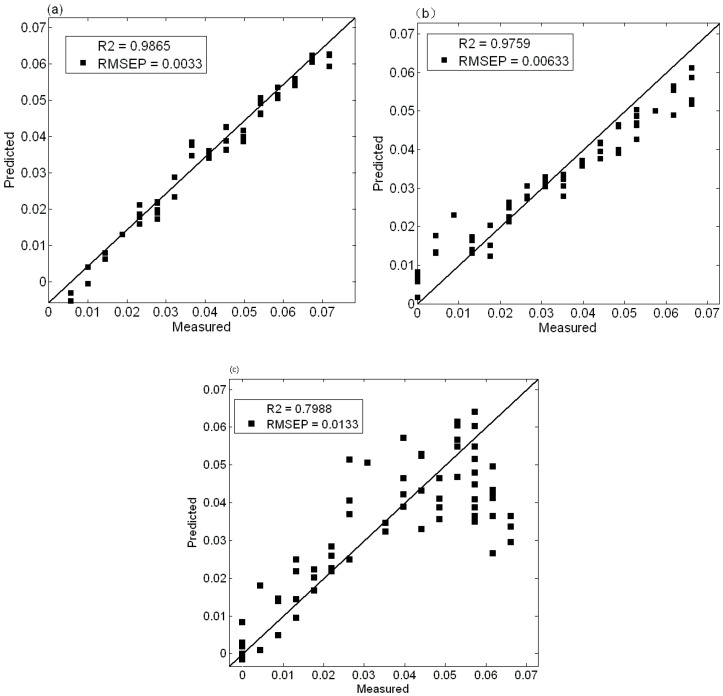
PLS modeling and prediction of soil nitrogen in three groups; (**a**) Strict pretreatment soil samples; (**b**) grinding and drying soil samples; (**c**) drying soil samples.

**Figure 4:**
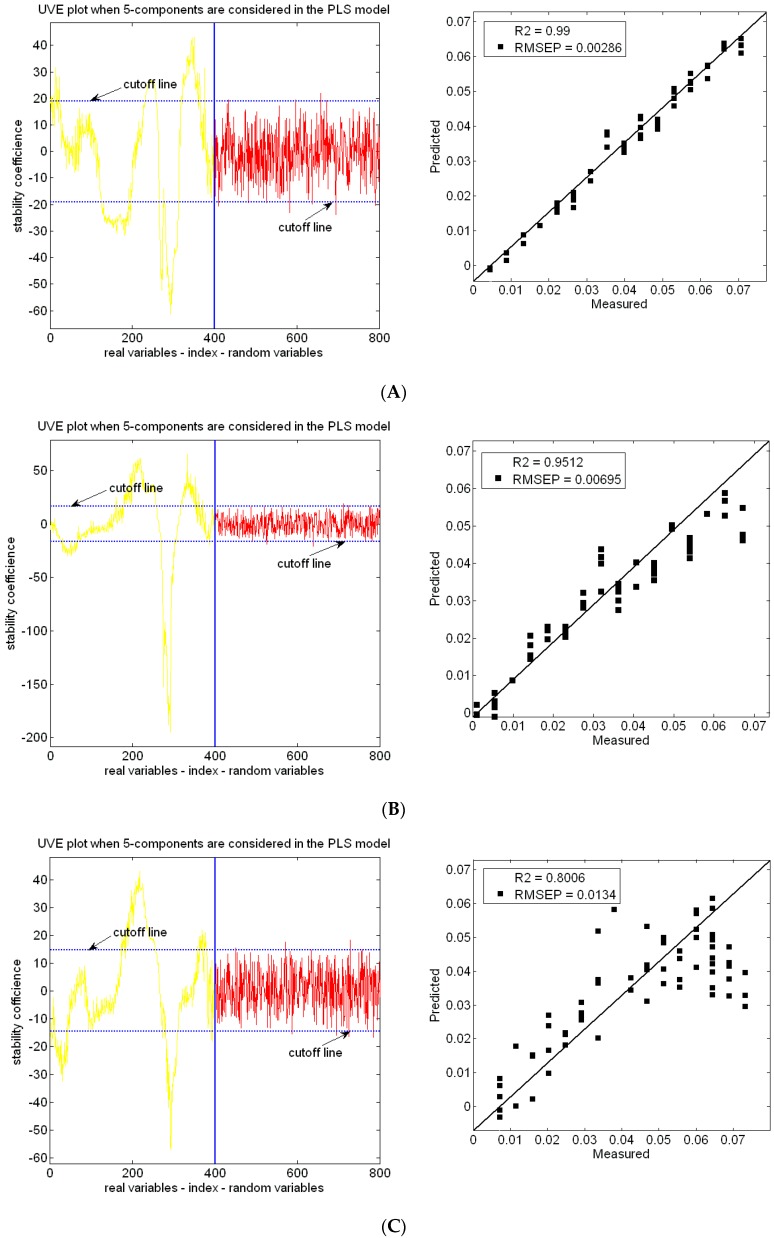
Modeling and prediction of soil nitrogen with UVE method: (**A**) Strict pretreatment soil samples; (**B**) grinding and drying soil samples; (**C**) drying soil samples.

**Figure 5:**
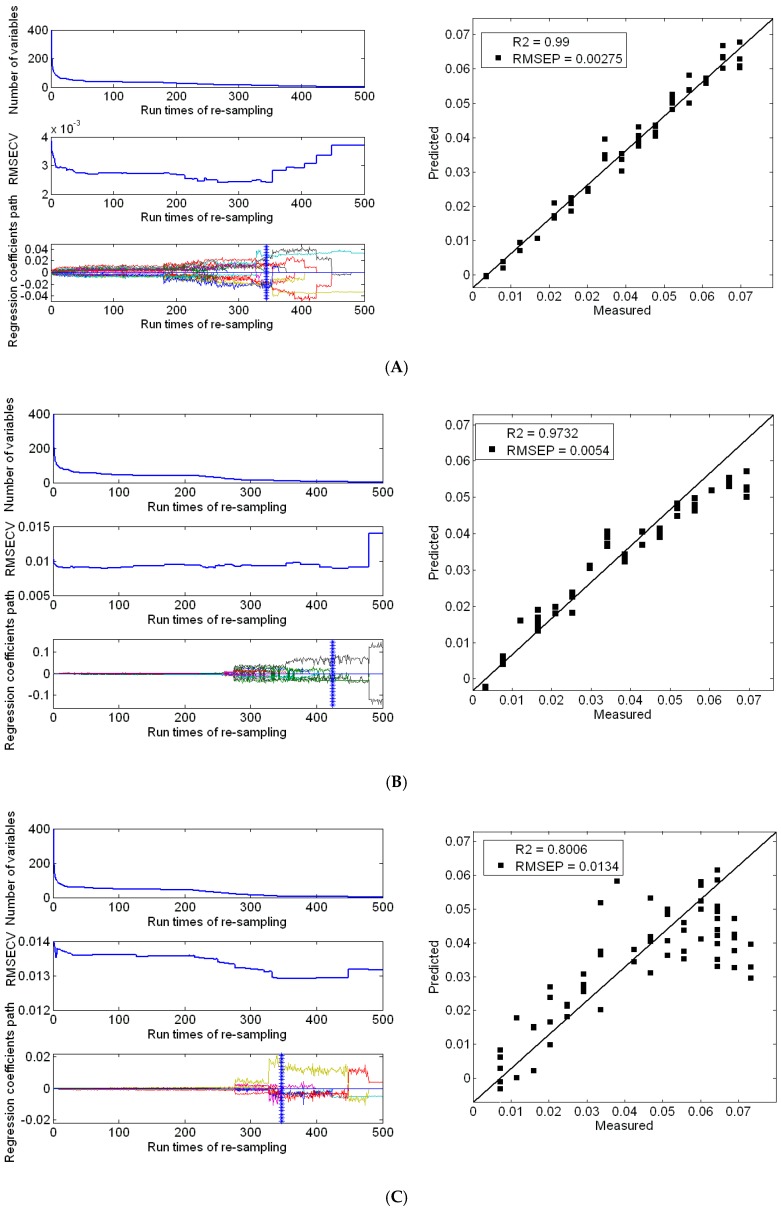
Modeling and prediction of soil nitrogen by CARS: (**A**) Strict pretreatment soil samples; (**B**) grinding and drying soil samples; (**C**) drying soil samples.

In this paper, the full-spectrum spectral data after baseline correction and normalization are taken as the independent variables *X* and the nitrogen content are taken as the dependent variable *Y*. The optimal number of factors are determined by the minimum root mean square error of cross validation (RMSECV, root mean square error of cross validation). At the same time, the spectral matrix *X* and the concentration matrix *Y* are decomposed by PLS, UVE and CARS. The relationship between them are taken into account to obtain the optimal correction model. In the experiment, 160 soil samples are divided into 16 groups according to different nitrogen concentration. The calibration set and validation set are modeled according to the ratio of about 5:3. The 100 samples are used for calibration set and 60 samples are for the validation set. R is the determination coefficient and RMSEP (root mean error of prediction) is the root mean square error of prediction. The smaller the RMSEP value is, the better the predictive ability and the higher the precision are. As is shown in the Figure 3, Figure 4 and Figure 5, it can be concluded from the figures.

In Figure 3, the PLS model shows that the coefficient of determination is 0.9901 and the value of RMESP is 0.002915 of the soil samples with strict pretreatment. The coefficient of determination is 0.9759 and the RMESP value is 0.00633 of the soil sample after drying and grinding. The coefficient of determination is 0.7988 and the RMESP value is 0.0133 of the dried soil samples. 

Figure 4 expresses the stability coefficient of the variables for each wavelength in the UVE model. The dotted lines are cutoff lines, which are determined by the added random numbers. The variables between the two cutoff lines are considered to be uninformative variables that need to be eliminated. The coefficient of determination is 0.99 and the value of RMESP is 0.00286 in the first group. The coefficient of determination is 0.9512, and the RMESP value is 0.00695 in the second group. The coefficient of determination is 0.8006 and RMESP is 0.0134 in the third group.

Figure 5, it describes the variable selection process of CARS. RMSECV presents a decreasing trend, which indicates that the eliminated variables are useless. RMSECV then starts to increase, and it may eliminate useful variables. The coefficient of determination is 0.99 and the value of RMESP is 0.00275 in the first group. The coefficient of determination is 0.9732 and the RMESP value is 0.0054 in the second group. The coefficient of determination is 0.7950 and RMESP is 0.0132 in the third group.

Therefore, a series of strict pretreatment soil samples, including drying, grinding, sieving and pressing, have the best accuracy of nitrogen detection by near-infrared sensor. The soil samples after drying and grinding have higher precision, while the precision of the soil samples only after drying is the lowest. However, compared with other near infrared detection of soil nitrogen, the accuracy of this experiment has been greatly improved. It is proved that the method is effective and reliable for the detection of nitrogen in soil after strict pretreatment.

### 3.3. Non-Linear Modeling and Analysis

**Figure 6:**
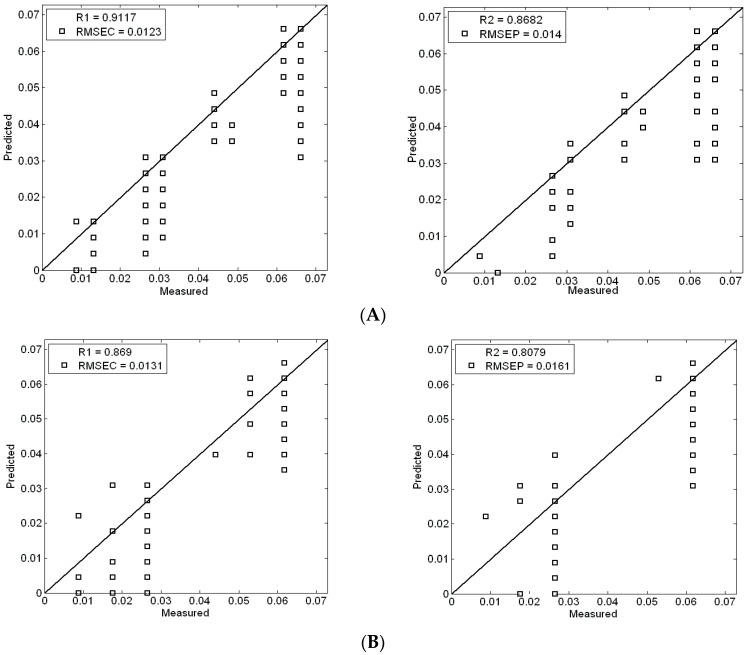

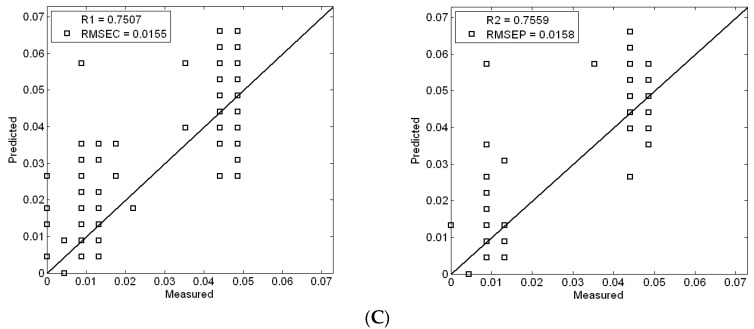
Modeling and prediction of soil nitrogen with LS- SVM: (**A**) strict pretreatment soil samples; (**B**) grinding and drying soil samples; (**C**) drying soil samples.

In order to further study the relationship between soil reflectance spectrum and soil pretreatment, and eliminate the influence of different pretreatment, three groups of data are analyzed by Partial least squares support vector machine (LS-SVM). The calibration set and validation set are modeled according to the ratio of about 5:3. The results of the analysis are shown in Figure 6.

Figure 6 presents the support vector machine modeling and prediction results. The modeling coefficient of determination is 0.9117 and the value of RMESC is 0.0123. The prediction coefficient of determination is 0.8682 and the value of RMESP is 0.014 in the first group. The modeling coefficient of determination is 0.869 and the RMESC value is 0.0131. The prediction coefficient of determination is 0.8079 and the value of RMESP is 0.0161 in the second group. The modeling coefficient of determination is 0.7507 and the value of RMESC is 0.0155. The prediction coefficient of determination is 0.7559 and the value of RMESP is 0.0158 in the third group. This shows that the use of nonlinear support vector machine method is not particularly desirable. Nevertheless, on the whole, the first set of pretreated soil samples has the best accuracy.

### 3.4. Comparison of the Three Linear Modeling Methods

In order to study the effect of soil pretreatment on the detection of soil nitrogen by near infrared, this paper used different pretreatments on three groups of soil samples and partial least squares regression, uninformative variable elimination and competitive adaptive reweighted algorithm methods. The data partitioning statistical results of the three groups of soil samples with conventional methods are represented in Table 1.

As can be seen from Table 1, 160 samples were tested in the experiment (three soil samples were damaged in the first group). The data set is divided into two subsets using the sample set partitioning based on joint *X*-*Y* distances (*SPXY*) method with a ratio of 5:3. Therefore there were 100 samples in the calibration set for training the samples and establishing the model, and the remaining 60 samples in the validation set are applied for testing the samples and verifying the performance of the model. The average values of the predicted values of the three soil samples are 0.0036, 0.0035 and 0.0037, respectively. The standard deviation values of three groups of soil samples are 0.0191, 0.0200 and 0.0214, respectively, which shows that the more stringent the soil pretreatment is, the smaller the standard deviation value is, and the better the detection effect is. On the other hand, the correlation coefficients R and RMSECV of the three modeling methods are shown in Table 2.

From Table 2, it is concluded that there are high correlation coefficients between the three kinds of correction models and prediction models, and the RMSEC and RMSEP values are small. The results shows that the pretreatment method has a good effect on the detection of soil nitrogen. Compared with the first group, the three soil sample models after drying and grinding pretreatment are worse than others. The possible reasons are that the particle size, the texture of the sample surface and the degree of compaction of the soil samples that are not sieved and pressed have an influence on the accuracy and expression of their reflectivity. The correlation coefficient of the soil samples after drying is only about 0.8 and the results are unsatisfactory, indicating that the surface irregularity and roughness of the soil samples cause diffuse reflectance, which resulted in great difficulties in the detection of soil nitrogen by near infrared sensor.

## 4. Conclusions

In order to study the effect of soil pretreatment on the detection of soil nitrogen by using near infrared sensors, this paper used (CO(NH_2_)_2_) to produce nitrogen solutions with 16 different concentrations. First, the different concentrations of organic nitrogen solution were fully mixed and stirred with soil. Second, the soil samples were divided equally into three groups and each group had 16 parts. Third, the three groups of soil samples were pretreated in different ways. The experiments uses partial least squares, no informative variable elimination method and competitive adaptive reweighted algorithm and partial least squares support vector machine. It is concluded that the soil samples with strict pretreatment have the best nitrogen detection accuracy by using a near-infrared sensor. The soil samples after drying and grinding have higher precision and the precision of the soil samples after only drying is the lowest. The main conclusions are as follows:

First, according to the reflectance characteristics of soil samples with different concentrations, when (CO(NH_2_)_2_) is not added to the soil, the spectral reflectance near the 925 nm and 1410 nm bands decreases significantly. With the increase of urea concentration, the spectral reflectance decreases gradually in the 1485 nm and 1640 nm bands. The reflectance of the third group is significantly worse than that of the former two groups. The reflectance of soil nitrogen is small and the absorption is not obvious on the 925 nm and 1410 nm bands. With the increase of nitrogen concentration, the spectral reflectance from 1420 nm to 1475 nm drops and fluctuates significantly in the characteristic band from 1475 nm to 1520 nm.

Second, among the different mathematical modeling methods, on the one hand, the correlation coefficients of PLS, UVE and CARS are good, and the RMSECV values are low in the first group of experiments. The second group has higher precision and the precision in the third group is the lowest. On the other hand, the use of nonlinear support vector machine method is not particularly desirable. However, on the whole, the first set of pretreated soil samples had the best effect.

Third, according to the soil pretreatment effect, the experiment with strict pretreatment reduces soil moisture content, soil particle size, soil surface roughness and other negative impacts. Compared with other soil nitrogen near infrared detection studies, the accuracy of this experiment has been greatly improved. Therefore, the experimental effect of the first group is significantly better than that of the latter two groups. Using this method, the detection of nitrogen content in soil by using a near infrared sensor was reliable and accurate.

## Figures and Tables

**Table 1:** Descriptive statistics for sample measurements.

Group	Dataset	Number	Average	Std
1	cal	100	0.0317	0.0219
	val	57	0.0363	0.0191
2	cal	100	0.0329	0.0207
	val	60	0.0335	0.0200
3	cal	100	0.0322	0.0198
	val	60	0.0378	0.0214

**Table 2:** Comparison of three mathematical modeling methods.

Group	Model Method	R1 of the Correction Set	R2 of the Prediction Set	Calibration Set RMSEC	Prediction Set RMSEP
Strict pretreatment soil samples	PLS	0.9901	0.9865	0.00292	0.00330
	UVE	0.9937	0.9900	0.00233	0.02860
	CARS	0.9949	0.9900	0.00210	0.00275
grinding and drying soil samples	PLS	0.8548	0.9759	0.01071	0.00633
	UVE	0.9176	0.9512	0.00821	0.00695
	CARS	0.9202	0.9732	0.00808	0.00540
drying soil samples	PLS	0.7872	0.7988	0.01216	0.01330
	UVE	0.7860	0.8006	0.01219	0.01340
	CARS	0.8169	0.7950	0.01137	0.01320
